# P-552. Analysis and Spatiotemporal Distribution of Infectious Disease Early Warning Signals During a Major Flooding Event: New South Wales and Queensland 2022 Floods

**DOI:** 10.1093/ofid/ofaf695.767

**Published:** 2026-01-11

**Authors:** Raina MacIntyre, Samsung Lim, Abrar Chughtai, Adriana Effie Notaras

**Affiliations:** The Kirby Institute, University of New South Wales, Sydney, New South Wales, Australia; School of Civil and Environmental Engineering, Sydney, New South Wales, Australia; School of Population Health, University of New South Wales, Sydney, New South Wales, Australia; The Kirby Institute, University of New South Wales, Sydney, New South Wales, Australia

## Abstract

**Background:**

Floods and climate-related disaster events have been associated with increased risk of certain infectious diseases. Japanese encephalitis virus (JEV) appeared unexpectedly for the first time on the mainland of Australia in 2022 amid major floods and may be associated with such events. Australian-based research investigating the association between recent and major flood events and infectious disease epidemiology remains limited. Early warning surveillance systems (EWSS) may provide early indicators of infectious disease threats after extreme weather events.

**Figure:**
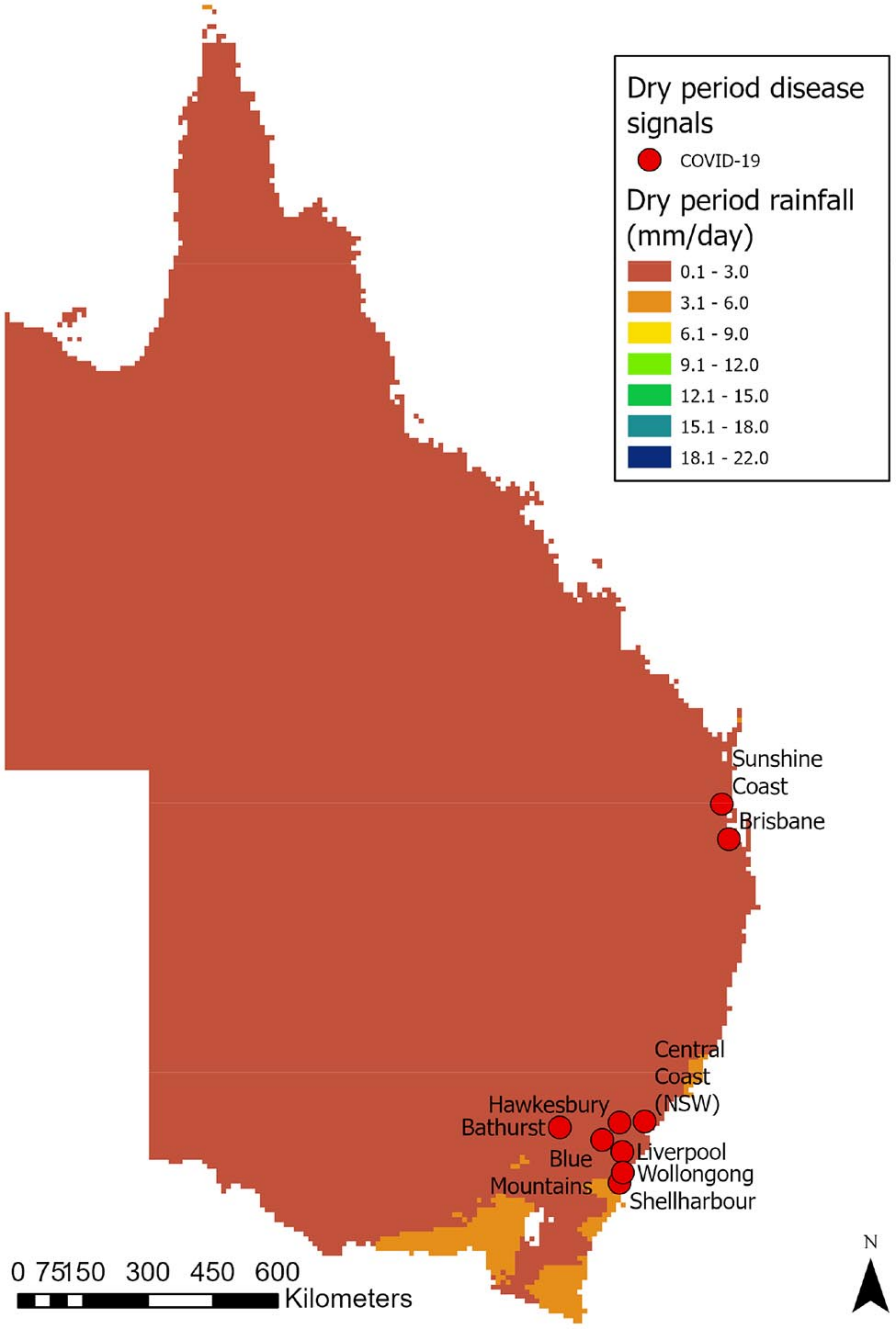
Disease signals and rainfall level during the dry period Data from May 1 2021 to Jul 31 2021 was used for the dry period

**Figure:**
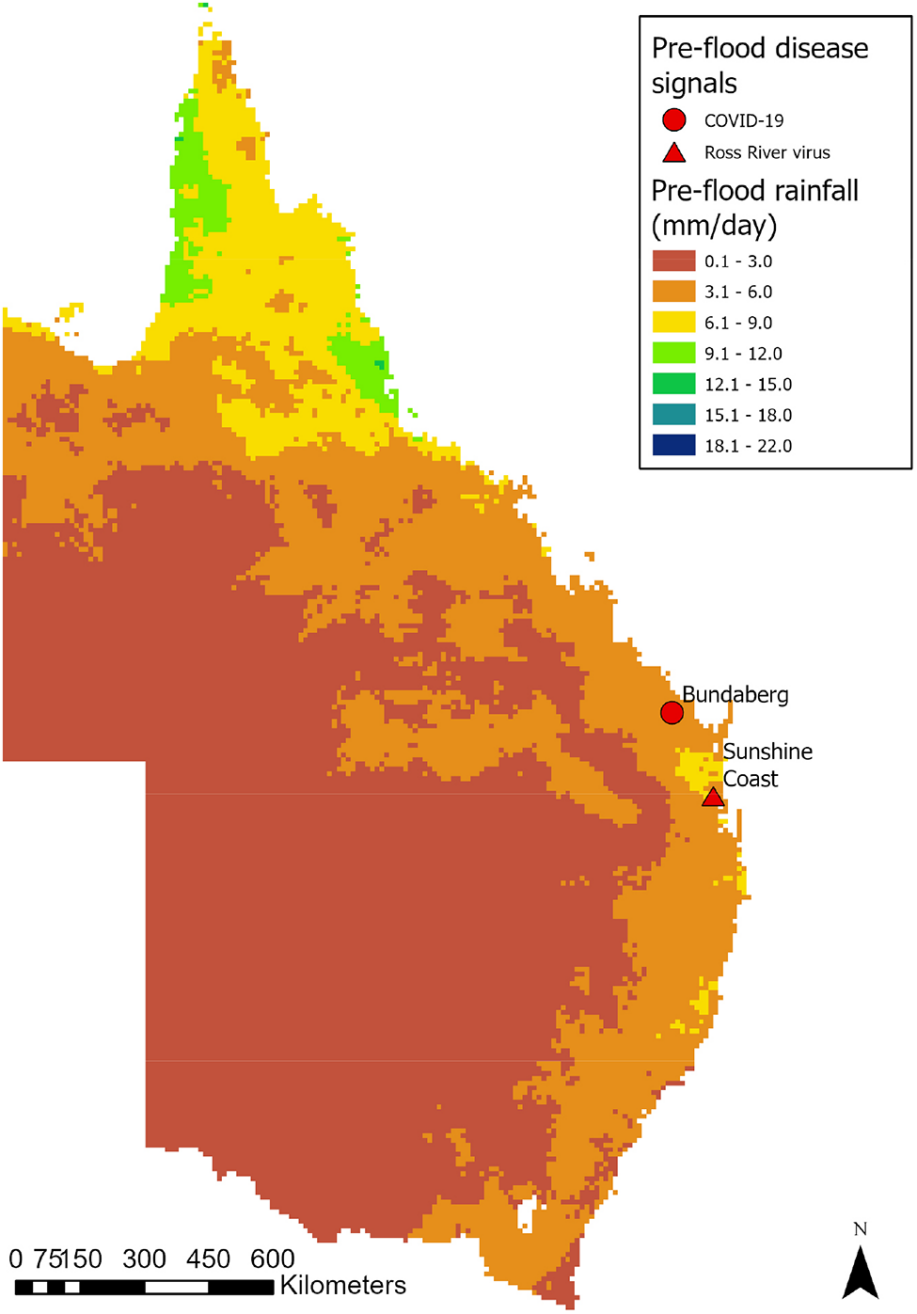
Disease signals and rainfall level during the pre-flood period Data from Nov 22 2021 to Feb 21 2021 was used for the pre-flood period

**Methods:**

We extracted retrospective disease data from the EPIWATCH open-source intelligence-based (OSINT) surveillance system before, during and after a flood period to generate disease signals that included local government area (LGA) data where possible. We analyzed the spatiotemporal distribution of disease signals and rainfall water level over the major 2022 flood period for all of NSW and QLD. We included three time periods for disease signal and rainfall data analysis in addition to the mid-flood period, including a ‘dry’ period as the control, a pre-flood and post-flood period, all covering three months.

**Figure:**
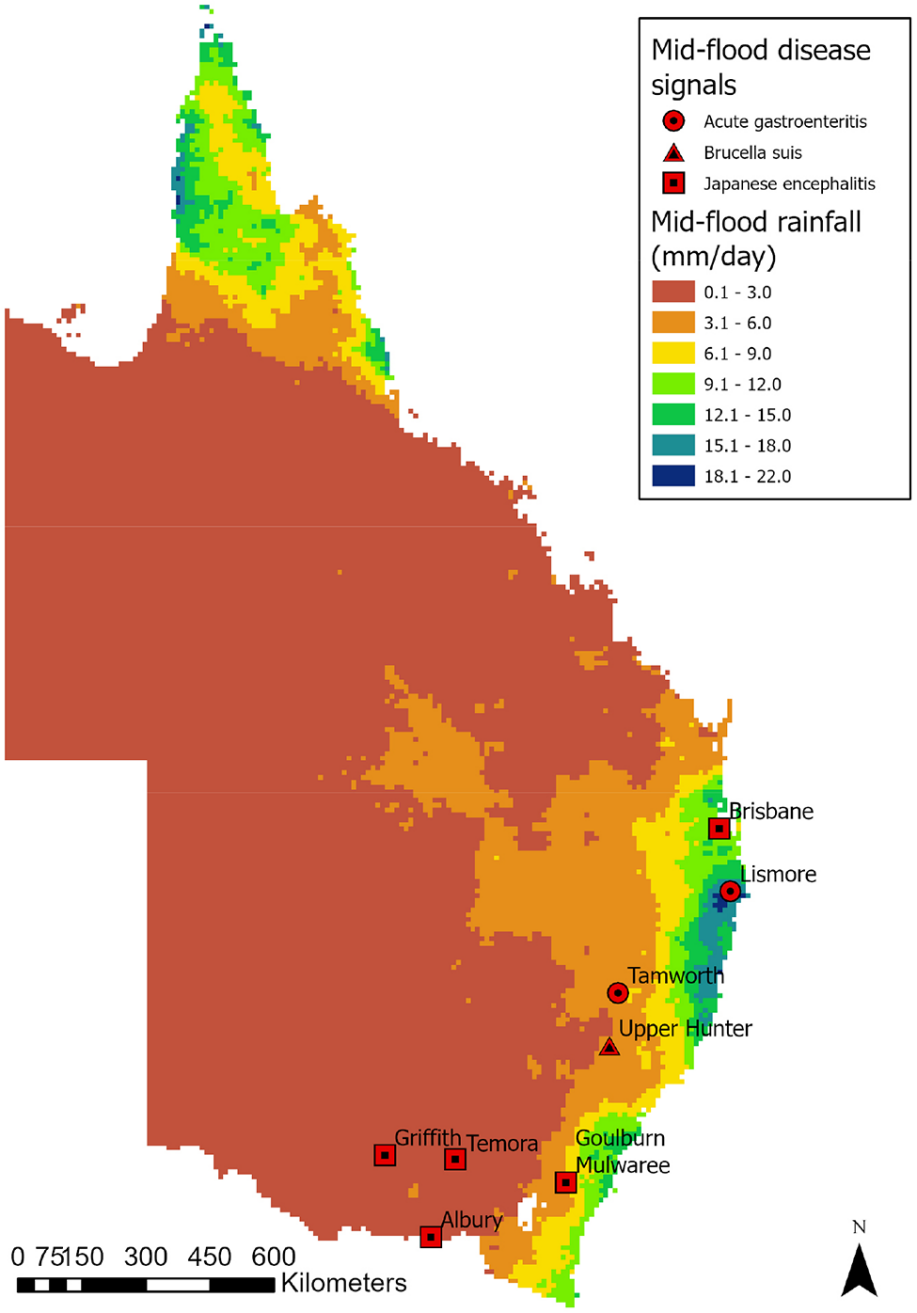
Disease signals and rainfall level during the mid-flood period Data from Feb 22 2022 to Apr 5 2022 was used for the mid-flood period, which corresponds with heavy rain and flooding events

**Figure:**
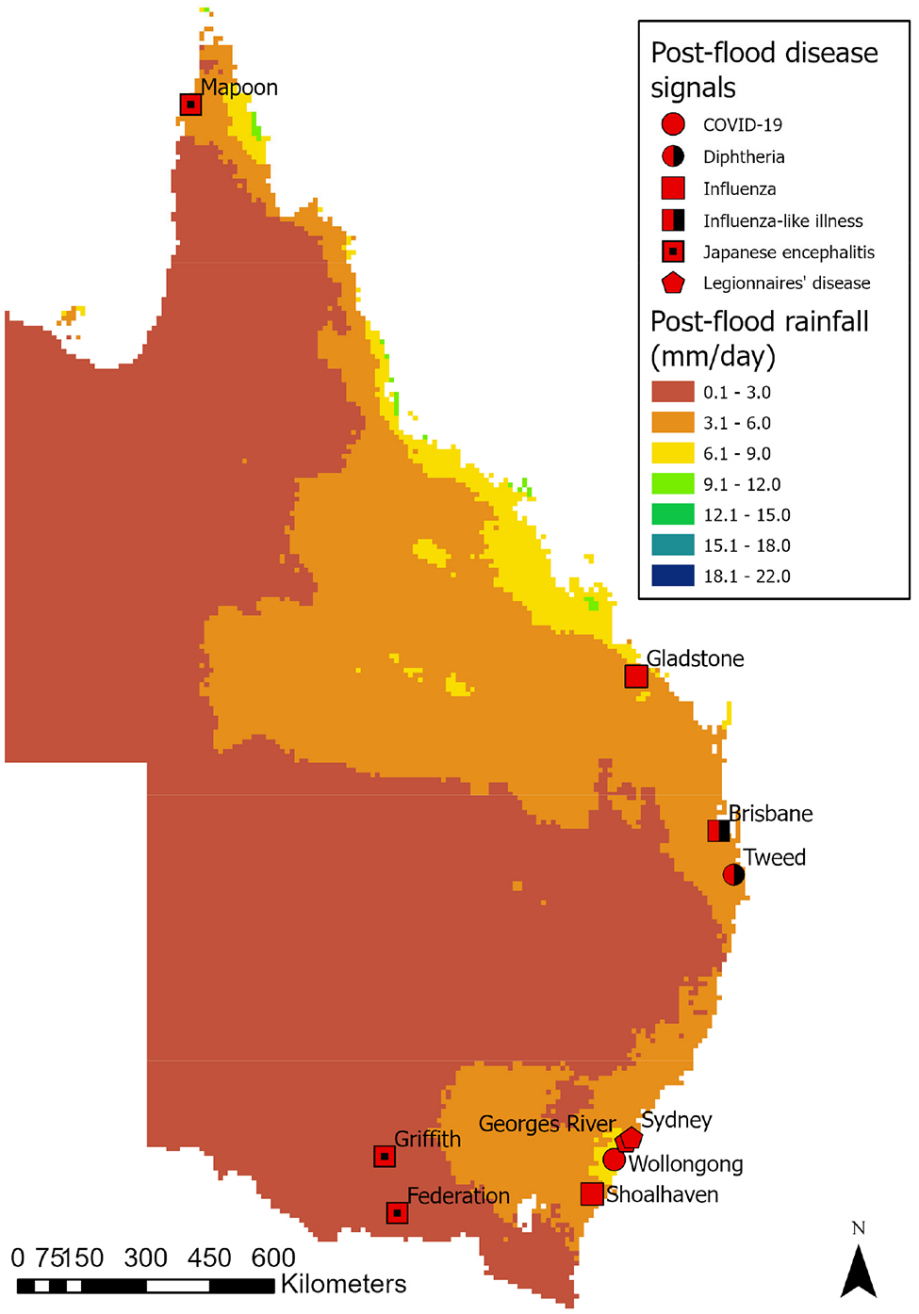
Disease signals and rainfall level during the post-flood period Data from Apr 6 2022 to Jul 5 2022 was used for the post-flood period

**Results:**

A total of 347 signals were included in the analysis. Signals were lowest (30) in the pre-flood period (30), increasing during the mid (80) post-flood periods (91), with animal disease signals peaking in the mid-flood period (14). Acute gastroenteritis in humans, pythiosis in animals and JEV in both humans and animals appeared to be associated with high levels of rainfall. The spatiotemporal distribution of signals suggests that outbreaks predominantly appeared in areas with increased rainfall during the mid and post-flood periods.

**Conclusion:**

Open-source *s*ignals of disease in both animals and humans seemed to correlate with major flooding events. In countries with increased risk of major flooding events, EWSS can assist with preparedness and response to diarrheal diseases and emerging infectious diseases, such as JEV. This study is among a limited number of papers investigating EWSS data and weather events in an Australian context. The addition of EWSS as an adjunct to formal surveillance can strengthen preparedness and response for the health effects of extreme weather events.

**Disclosures:**

Raina MacIntyre, MBBS Hons 1, FRACP, FAFPHM, M App Epid, PhD, National Health and Medical Research Council (NHMRC), Australia: Grant/Research Support|Sanofi: Grant/Research Support

